# Impact of COVID-19 pandemic on waste management

**DOI:** 10.1007/s10668-020-00956-y

**Published:** 2020-08-26

**Authors:** Samuel Asumadu Sarkodie, Phebe Asantewaa Owusu

**Affiliations:** Nord University Business School, Bodø, Norway

**Keywords:** COVID-19 and waste management, COVID-19 pandemic, SARS-COV-2, Social distancing measures, COVID-19 and environment, Novel coronavirus

## Abstract

The containment of the spread of COVID-19 pandemic and limitations on commercial activities, mobility and manufacturing sector have significantly affected waste management. Waste management is critical to human development and health outcomes, especially during the COVID-19 pandemic. The invaluable service provided by the waste management sector ensures that the unusual heaps of waste that poses health risks and escalate the spread of COVID-19 is avoided. In this study, we assess the impact of COVID-19 pandemic on waste management by observing lockdown and social distancing measures. We found that the quantity of waste increased across countries observing the social distancing measure of staying at home. The intensification of single-use products and panic buying have increased production and consumption, hence thwarting efforts towards reducing plastic pollution. However, several countries have thus far instituted policies to ensure sustainable management of waste while protecting the safety of waste handlers.

## Introduction

**Fig. 1:**
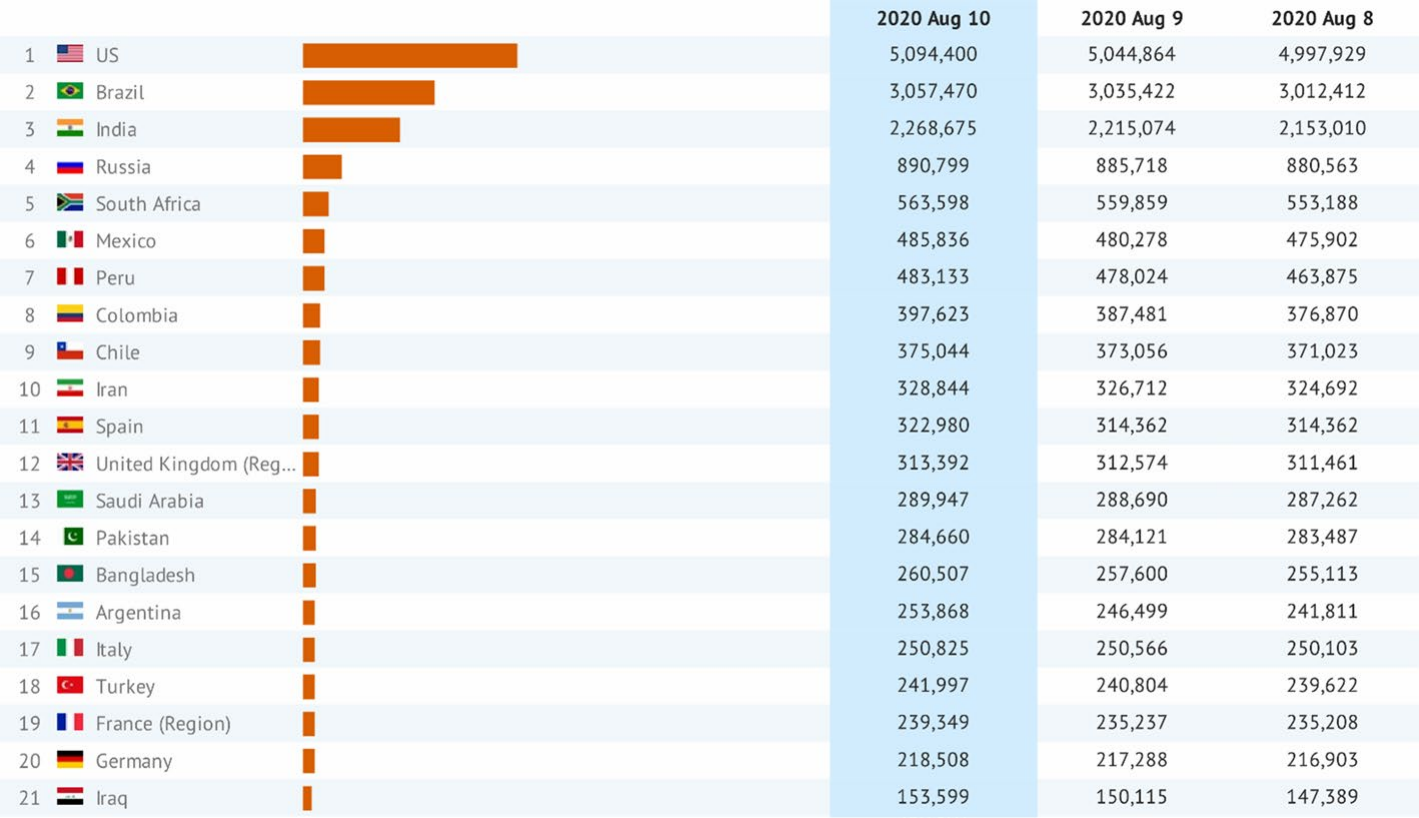
Global distribution of confirmed COVID-19 (Top 21 countries). *Data source*: Lauren (2020)

Currently, there are over 20.1 million global confirmed cases and ~ 742 thousand deaths across the globe. The top ten countries depicted in Fig. 1 with reported cases include the USA (5,094,400 persons), Brazil (3,057,470 persons), India (2,268,675 persons), Russia (890,799 persons), South Africa (563,598 persons), Mexico (485,836 persons), Peru (483,133 persons), Colombia (397,623 persons), Chile (375,044 persons) and Iran (328,844)—with total confirmed cases surpassing ~ 13.95 million [est. August 10, 2020 at 18:00 GMT] (Lauren 2020). Despite income group (low, middle and higher income), the COVID-19 pandemic has exposed several lapses and limitation of the current socio-economic, health and environment-related sectors across countries (Owusu and Asumadu 2020). Though the COVID-19 pandemic is reported to have reduced air pollution and environmental-related noise and improved biodiversity and tourist sites, however, the impact of stay-at-home and preventive measures on waste management is alarming (Box 1). Due to the stockpiling of gloves, gowns, masks and other protective clothing and equipment, there appears to be a waste emergency due to the unusual production of waste from both households and health facilities (Ma et al. 2020). Failure to properly manage the waste generated from health facilities and households may escalate the spread of COVID-19 via secondary transmission. The potential rampant dumping, open burning and incineration could affect air quality and health outcomes due to the exposure to toxins (WHO 2020). Thus, there exists a challenge of managing unusual waste sustainably using available waste facilities while reducing air pollution, preventing secondary viral transmission and mitigating potential health risk (UNEP 2020a). Besides, there could be serious consequences for developing countries without standard waste management technologies and waste emergency policies to curb the pandemic.

**Box 1:**
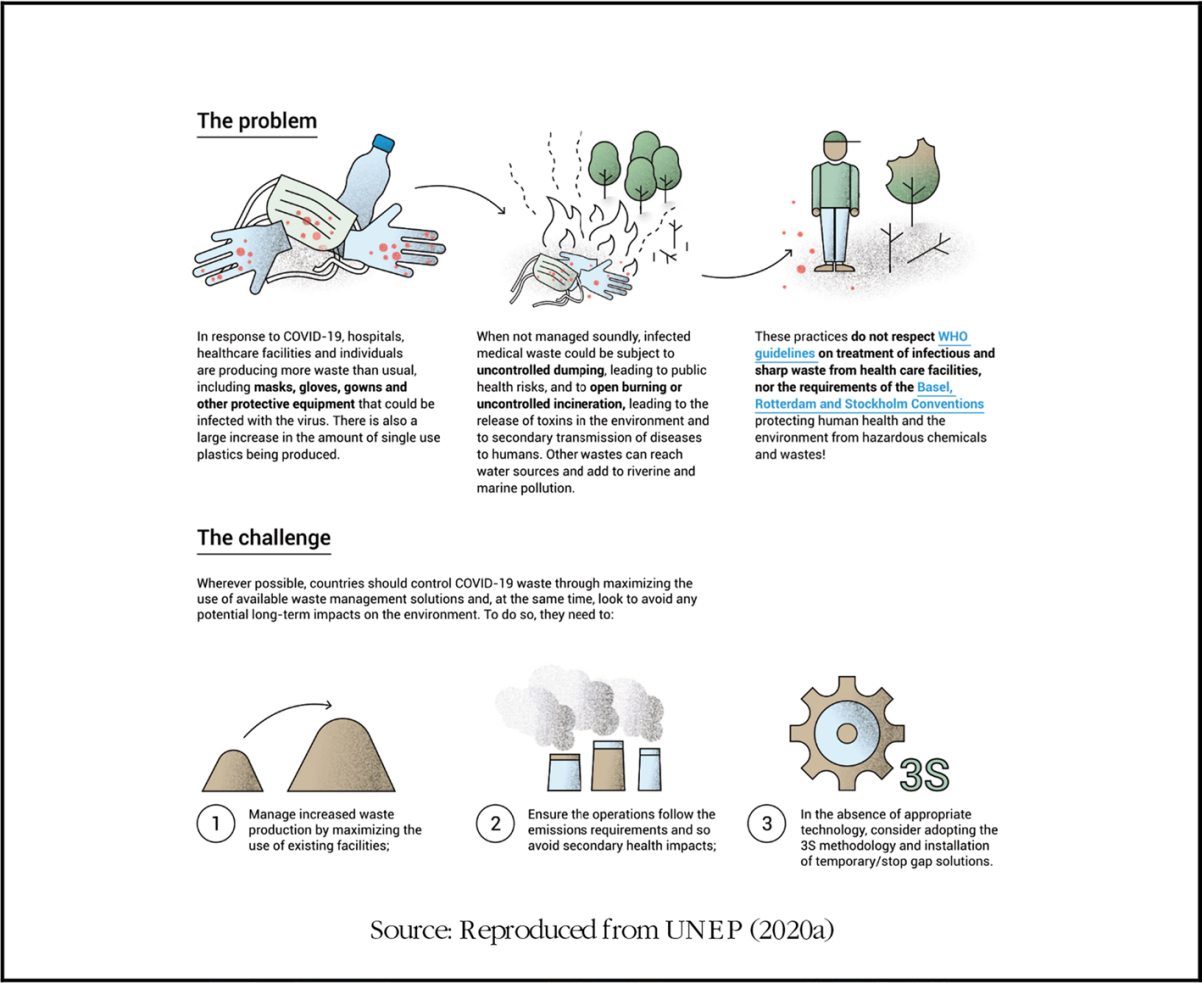
Assessing the challenge of sustainable waste management due to COVID-19

As such several guidelines have been proposed ranging from (Cristina 2020): (1) proper management of municipal solid waste using personal protective equipment, safety practices and administrative and engineering controls. (2) managing COVID-19 contaminated medical waste as regulated medical waste. (3) recycling of waste using safety practices that prevent infection and cross-contamination. (4) proper management of wastewater using ultraviolet irradiation for inactivation, and peracetic acid and hypochlorite for oxidation. Thus, waste management is an essential public service required to contain the spread of COVID-19 (UNEP 2020b). Here, we assess the impact of COVID-19 pandemic on waste management across the globe. Due to data limitation, we present both qualitative and quantitative data reported in government web platforms, development cooperations, news and published articles.

## Stylized facts

Before the COVID-19 pandemic, the world was already facing challenges in the waste management sector, where over two billion people lack access to waste collection whereas over three billion people lack access to waste disposal (UN-Habitat 2020). Hence, the emergence of the COVID-19 pandemic and its corresponding social distancing measures amplify the already burdened sector (Box 2). The shutdown of hotels, restaurants, and other food-related services due to lockdown and social distancing measures have driven outdoor rats indoors. Due to less garbage on streets, there are reports of a 50% increase in indoor rat infestation in urban areas in Canada (SWR Staff 2020). The growing health concern of rats’ infestation is their ability to carry disease-causing pathogens such as Escherichia coli and salmonella, and transmit to humans (Nkogwe et al. 2011). Thus, household waste requires proper management techniques to keep rats away from buildings and homes.

**Box 2:**
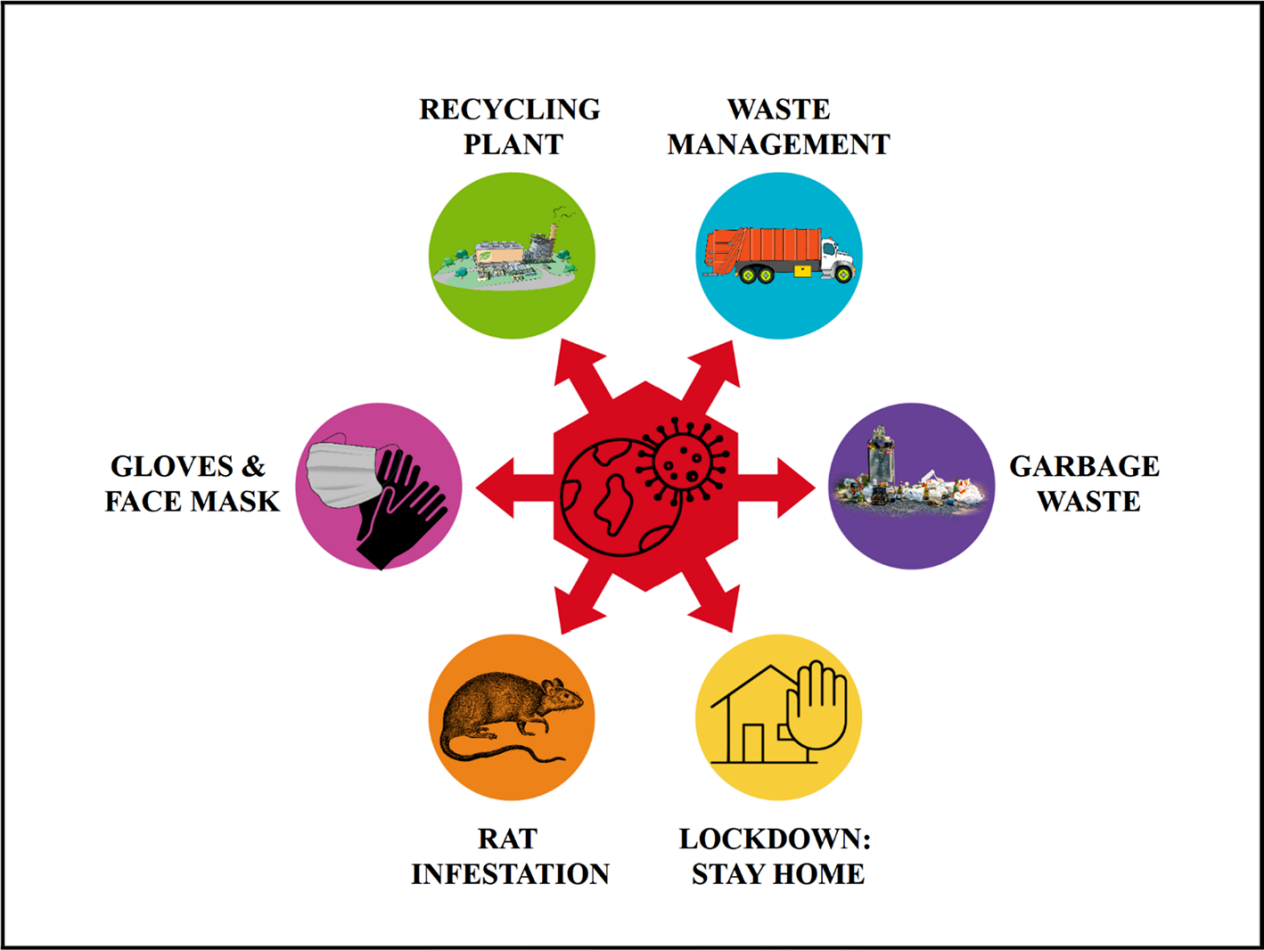
Interaction between waste adaptation and COVID-19 Pandemic

The onset of the COVID-19 pandemic led to the institution of distancing measures that triggered panic buying of food, toilet papers, face masks, gloves, cleaning products and 70% alcohol-based hand sanitizers (Sarkodie and Owusu 2020a). During this period, shopping of basic protective equipment, products and groceries grew by over 20% in one Supermarket alone (Reconomy 2020). This panic buying increased the disposal of perishable products and leftovers, which ultimately generated tonnes of waste. In just 15 countries in Africa alone, the total face masks per day are reported as 586,833,053 based on 80% acceptance rate and an average of 2 face masks daily per capita (Nzediegwu and Chang 2020). However, the question remains how these tonnes of waste can properly be disposed of with the limited technological innovation and recycling equipment in Africa.

The lockdown period due to social distancing measures to contain the spread of COVID-19 is reported to have increased the use of plastics, a situation that has policy implications (Klemeš et al. 2020). The lifecycle of plastics from cradle (extraction) to grave (disposal) is dangerous and have an environmental cost. Refineries for plastic are reported to increase the exposure to toxic chemicals leading to increased health outcomes such as mortality, morbidity and disability-adjusted life-years. Thus, increasing use of plastics during the lockdown and stay-at-home measures serve as a conduit for contamination between pathogens of animal and human origin—which increase the spread of diseases (Perry 2020). There are reports of over million synthetic face masks and gloves disposed on sidewalks already polluting cities (Emily 2020).

In Hong Kong, face masks are reported to have piled up at nature trails and beaches due to improper disposal in waterways, which disrupts the marine environment. Life below water is disrupted as marine habitat wrongly conceives the non-biodegradable plastics (polypropylene) from face masks as food (Farah 2020). Thus, improper disposal of face masks to the aquatic environment poses a great threat to both wildlife and marine life.

Besides, the COVID-19 pandemic has affected the recycling market due to the institution of social distancing measures like lockdown, hence affecting livelihoods. Due to low oil price and demand, the competitiveness of recycled plastics has declined, hence affecting the price of virgin plastics (Silpa 2020). A temporary ban on cross-border movements affects developing countries that depend on foreign technology for waste recycling activities, and hence, most of the waste generated during the pandemic is disposed of rather than recycled.

## Medical waste assessment

Sustainable management of medical waste is problematic and amplified, especially in emergencies like the COVID-19 pandemic (Box 3). Due to the novelty of the global pandemic, modification to existing waste facilities to control the unusual medical waste and its associated viral spread effect requires adequate information on the amount of medical waste generated, hot spots for waste generation and available treatment facilities. On account of potential rapid expansion volumes of medical waste, several technical know-how on sorting, segregation, transport, storage and sustainable waste management technologies are required to maximize existing infrastructures to accommodate the emergency (Sharma et al. 2020). Improper management of medical waste has the potential to expose patients, health workers and waste managers to injuries, infections, toxic consequences and air pollution (Mihai 2020). The different forms of medical waste and its derivatives include non-hazardous waste, pathological waste, radioactive waste, infectious waste, chemical waste, cytotoxic waste, sharps waste and pharmaceutical waste (WHO 2018). The global pandemic has led to an unusual amount of reported medical waste. For example, the COVID-19 pandemic in China is reported to have increased medical waste from personal protective equipment like gloves, face masks and eye protection due to a surge in personal protective equipment and immediate disposal after use (Ma et al. 2020). Due to the overwhelming surge in daily waste (i.e. over 240 metric tonnes) and increasing levels of hospital medical waste by sixfold, it is reported that the influx of COVID-19 patients led to the construction of waste plants and deployment of 46 mobile waste treatment facilities in China (Calma 2020). In Barcelona, medical waste such as overall, face masks and gloves increased by 350%—generating about 1,200 tonnes of medical waste compared to the usual waste of ~ 275 tonnes (ACR + 2020).

**Box 3:**
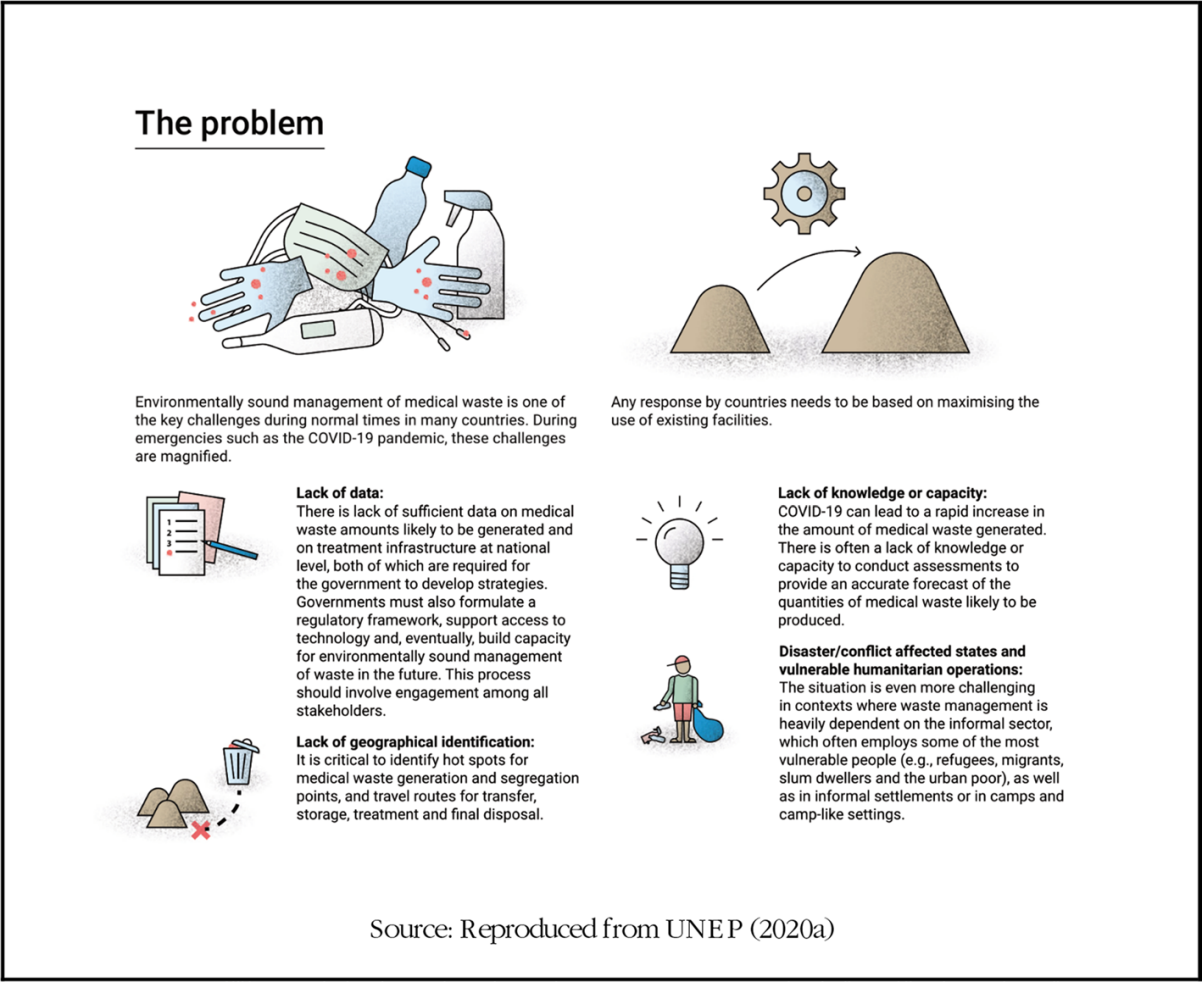
Challenges affecting measures to control unusual medical waste

## Non-medical and household waste

The institution of lockdown, stay-at-home policy and other preventive measures to contain the spread of COVID-19 saw an increase in production and consumption patterns of non-medical and household-related products such as masks, gloves, thermometers, sanitizers and cleaning products, toilet papers and foodstuffs (Box 4). Sudden lockdown and fear of the virus lead to the intensification of single-use products and panic buying (Sarkodie and Owusu 2020b). The unprecedented use of masks to reduce the exposure to COVID-19 is reported to have increased its production, hence increasing the global sales by US$166 billion (UN 2020). Due to the current role of protective equipment like disposable masks and gloves, the COVID-19 pandemic appears to have thwarted efforts to decline plastic pollution. To contain the spread of COVID-19, the World Health Organization projects a monthly global expenditure of 1.6 million plastic-based protective goggles, 76 million plastic-based examination masks and 89 million plastic-based medical masks (Andersen 2020). The daily production of plastic-based masks in February increased by 116 million (a dozen times higher than January) in China (W4C 2020). There are several reports of enormous plastic waste soaring from 1500 to 6300 tonnes daily in Thailand, owing to food products delivered to homes, whereas the UK saw 300% rise in illegal waste disposal during the lockdown period (Weforum 2020).

**Box 4:**
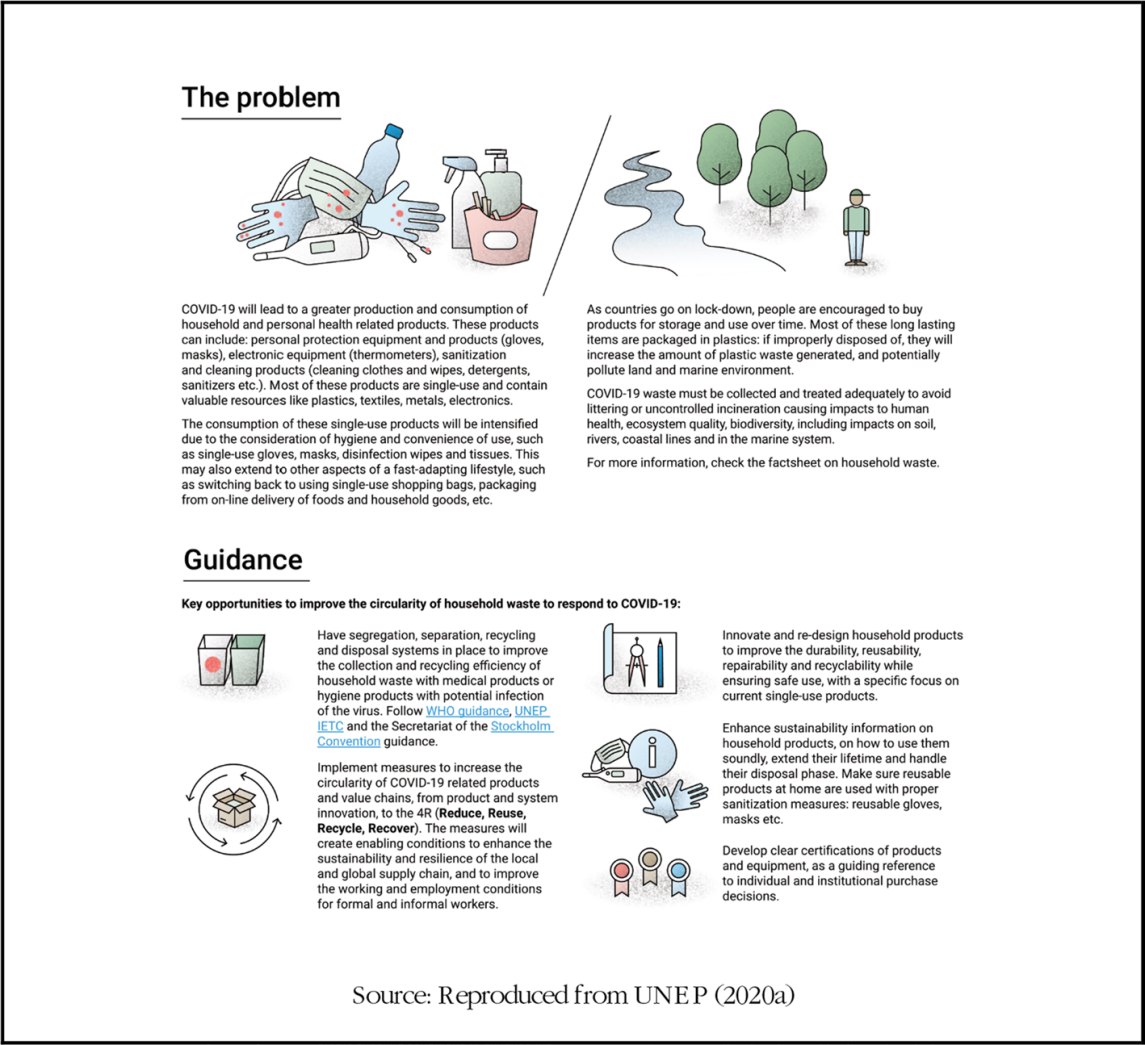
Non-medical waste assessment

The COVID-19 pandemic underscores the need for households to utilize a separate collection of waste (Box 5). In Milan (Italy), the institution of strict lockdown led to a decline in the total waste production by 27.5%, which includes 24.4% decline in residual waste, 20% reduction in paper and cardboard waste, 16.7% decrease in glass waste, 16.3% decrease in plastic and metal waste, 14.4% decline in household food waste and 80.5% reduction in commercial food waste (AMSA 2020). The reduction can be attributed to reduced waste production compared to other cities. However, recycling of waste increased by 1% compared to 2019 of the same period, whereas street bins declined by 38.2%. The month of confinement saw a 16.65% drop in municipal waste, thus from 282.3 thousand tonnes to 242 thousand tonnes in Catalonia (Spain). In Barcelona, waste generation fell by 25% due to mobile restrictions on tourist and commercial activities. Paper, glass, cardboard, organic and lightweight packaging declined by 20% whereas mixed waste declined by 12% (ACR + 2020).

**Box 5:**
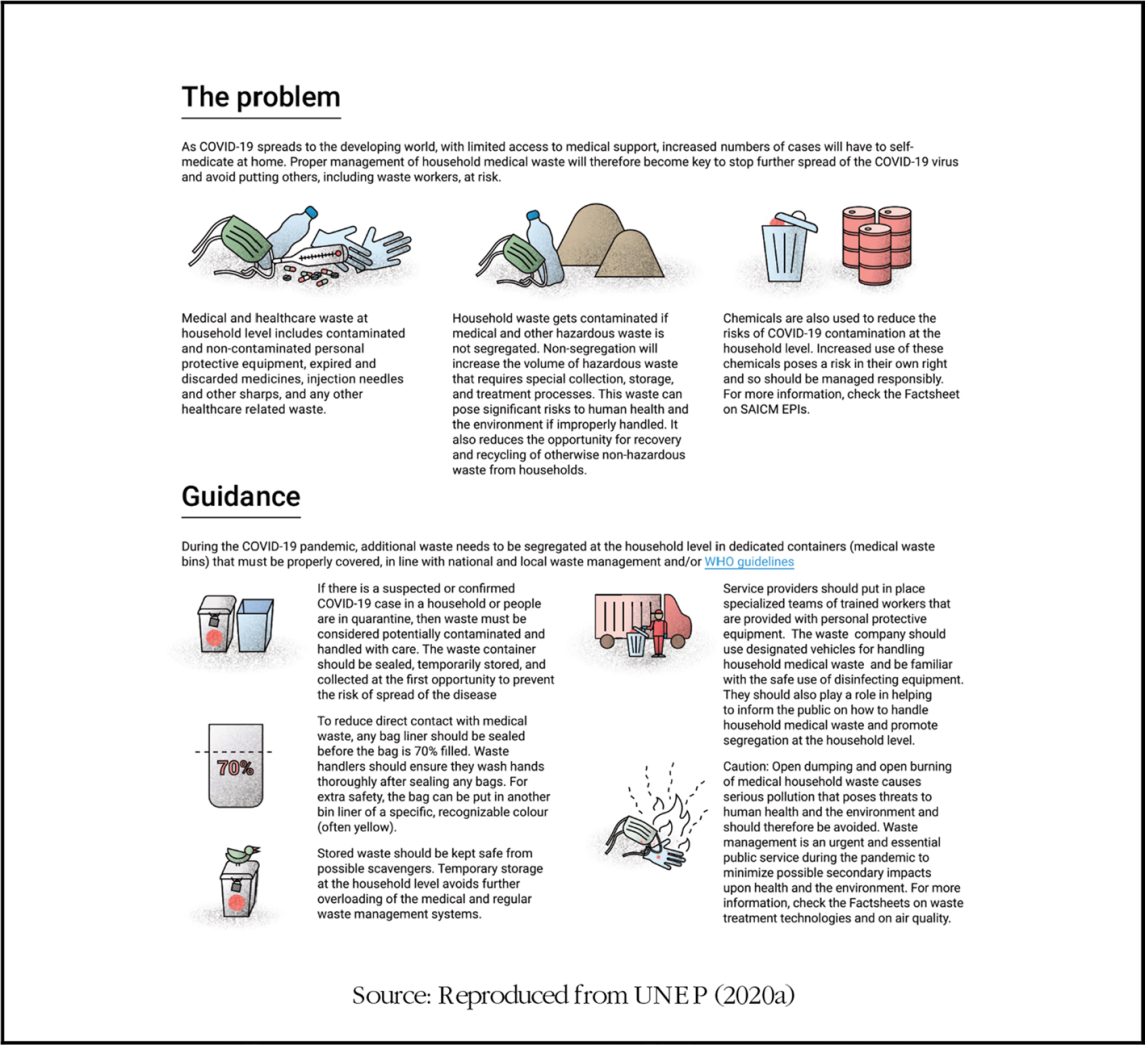
Household waste assessment

## Policy response and implication

Due to the overwhelming tonnes of waste generated during the lockdown, the Irish government announced a million euros funding ring-fenced to tackle the level of illegal dumping attributed to the COVID-19 crisis (DCCAE 2020).

Waste pickers whose livelihood depends on waste collection could no longer benefit and perform business-as-usual, due to the stringent social distancing measures. For example, waste pickers help in the collection of re-usable and recyclable dumped solid waste essential for the integration into economic production (Moreno-SÁNchez and Maldonado 2006). Hence, waste pickers play an essential role in achieving a circular economy, especially in developing countries. In Turkey, over 8,000 waste pickers were banned as part of COVID-19 containment measures; however, food aid and municipal shelters were provided (Hikmet 2020). Waste pickers are occupational risk group with more susceptibility to health conditions (Cruvinel et al. 2019); hence, the ban by the Turkish government prevented many waste pickers that would have been exposed to secondary transmission of COVID-19.

The COVID-19 pandemic has triggered a zero-waste approach that requires members of the EU to recycle waste between 70–80% while declining GHG emissions attributed to toxic waste disposal and incineration techniques (Zero Waste 2020). The zero-waste approach encompasses “the conservation of all resources by means of responsible production, consumption, reuse and recovery of products, packaging and materials without burning, and with no discharges to land, water or air that threaten the environment or human” (ZWIA 2018). The post-crisis offers lessons that waste management before the COVID-19 pandemic cannot be continued as business as usual but requires structural adjustments, hence accentuating the importance of transitioning from linear to a circular economy. This in effect navigates towards achieving zero-waste and zero-carbon-based economic development that has low waste management expenditure. Proper waste management within the COVID-19 pandemic ensures continuity and functionality of waste services and workers, the safety of waste service workers, adjustments of recycling services to incorporate safety measures that contain the spread in the collection, disposal and treatment of medical waste (ISWA 2020).

## Conclusion

With the increasing spread and impact of the COVID-19 pandemic on economic development and health outcomes, there is an urgent global call for waste management from households, medical facilities and toxic waste to be treated as essential public service. This will in effect mitigate the potential threats of COVID-19 pandemic on environmental sustainability and health outcomes. In line with the United Nations Environment Program of ensuring sustainable waste management, guidelines for containing the spread of COVID-19 through waste management include treatment of residual waste (tissues, handkerchiefs and similar organic and packaging waste) in incineration plants at a temperature near 1000-degree Celsius to ensure safe and complete destruction of the virus. COVID-19 has exposed the world to several environmental threats due to plastic pollution—attributable to unsustainable use of single-use plastics. Owing to the global adoption of personal protective equipment such as face masks, future research should aim at developing biodegradable and environmentally friendly protective gears including face masks, gloves, overalls, among others, to accelerate the agenda towards achieving sustainable production and consumption while reducing environmental costs.
